# *APOE* rare missense variants in Japan

**DOI:** 10.1002/alz.088676

**Published:** 2025-01-03

**Authors:** Akinori Miyashita, Norikazu Hara, Ai Obinata, Tamao Tsukie, Mai Hasegawa, Masataka Kikuchi, Kensaku Kasuga, Takeshi Ikeuchi

**Affiliations:** ^1^ Brain Research Institute, Niigata University, Niigata, Niigata Japan; ^2^ Department of Molecular Genetics, Brain Research Institute, Niigata University, Niigata, Niigata Japan; ^3^ Department of Computational Biology and Medical Sciences, Graduate School of Frontier Sciences, The University of Tokyo, Kashiwa, Chiba Japan

## Abstract

**Background:**

*APOE* is well recognized to be the most influential susceptibility gene for Alzheimer’s disease (AD). For the wild‐type allele, *e3*, it is known that the *e4* allele is a risk for AD, while the *e2* allele is protective. Recently, genetic analyses with Caucasians have reported the critical associations between *APOE* rare missense variants (RMVs) and AD, and their importance has been pointed out in terms of disease pathogenesis of AD. For example, the Christchurch (rs121918393, p.R136S [CGC > AGC]) and the Jacksonville (rs199768005, p.V236E [GTG > GAG]) variants can be mentioned. Since there have been no such analyses or reports yet in East Asians, including Japanese, we designed this study.

**Objective:**

To identify *APOE* RMVs in a Japanese population.

**Method:**

Through the Tohoku Medical Megabank Organization (ToMMo) with a cohort of healthy Japanese community residents, *APOE* variant data were obtained from 3,307 subjects (1,810 females, and 1,497 males; mean age 55.9 years [standard deviation 14.1]) over 20 years old. Rare variants with amino acid substitutions were selected from them. The human genome database, Ensemble Human (GRCh38.p14), was searched to obtain the allelic frequency data of identified RMVs by populations. We also investigated the association with *APOE* common alleles, *e2*, *e3*, and *e4*, in the background of RMV carriers.

**Result:**

A total of 176 variants were identified in and around *APOE* in an approximately 7.2 kb genomic region in the ToMMo cohort. Of these, 14 were RMVs, including those previously reported. Four RMVs were found to be very rare, which were not found in the 1000 Genomes or gnomAD exomes/genomes databases. The 13 RMVs were inferred to be under the wild‐type allele, *e3*, as background.

**Conclusion:**

Through this survey, it became clear that there are RMVs of *APOE* that are unique to the Japanese population. In the future, we will expand the analysis to a discovery project on a scale of tens of thousands of individuals, including our subjects (n = approx. 2,500) collected so far. For the newly discovered RMVs, we plan to investigate their effects on the metabolism of amyloid beta and tau proteins in relation to AD.

